# Sulfur-Dependent Disulfide Bond Disruption in Insulin Resistance: A Hypothesis

**DOI:** 10.34172/apb.025.46067

**Published:** 2025-08-03

**Authors:** Maher Monir. Akl, Amr Ahmed

**Affiliations:** ^1^Faculty of Medicine, Novosibirsk State University, Novosibirsk, Russia; ^2^The Public Health Department, Riyadh First Health Cluster, Ministry of Health, Riyadh, Saudi Arabia

## To Editor,

 Type 2 diabetes mellitus (T2DM), projected to affect over 700 million individuals by 2045, is conventionally attributed to peripheral insulin resistance driven by obesity, inflammation, and impaired insulin signaling.^1^ We propose the Sulfur Insulin Deformation Hypothesis, a conceptual framework suggesting that insulin misfolding, potentially arising from organic sulfur deficiency, may disrupt the integrity of insulin’s three disulfide bonds (A6-A11, A7-B7, A20-B19), thereby contributing to insulin resistance. This hypothesis proposes that T2DM may involve a sulfur metabolism disorder linked to mitochondrial dysfunction in intestinal epithelial cells, offering a novel perspective for further investigation into its pathogenesis. Insulin, a 51-amino-acid polypeptide comprising A (21 amino acids) and B (30 amino acids) chains, relies on three disulfide bonds formed through cysteine thiol oxidation to maintain its bioactive conformation and high-affinity binding to the insulin receptor (IR).^2,3^ In pancreatic beta cells, insulin biosynthesis begins with preproinsulin, which is cleaved to proinsulin and folded in the endoplasmic reticulum (ER). Protein disulfide isomerase (PDI) facilitates disulfide bond formation, a process dependent on cysteine availability.^4^ The A6-A11 bond, acting as a dynamic hinge, aligns critical residues (e.g., ValA3, TyrA19) for IR docking, while A7-B7 and A20-B19 stabilize the hydrophobic core.^5,6^ Disruption of these bonds, particularly A6-A11, may reduce IR affinity by 50-70%, potentially impairing phosphoinositide 3-kinase (PI3K)-Akt signaling and glucose transporter type 4 (GLUT4) translocation, which could contribute to hyperglycemia.^7,8^

 This hypothesis suggests that mitochondrial dysfunction in intestinal epithelial cells may impair the transsulfuration pathway (involving cystathionine β-synthase and γ-lyase), potentially reducing cysteine synthesis.^9^ Such cysteine deficiency could limit glutathione production, a critical antioxidant, by 30-73.8% in T2DM patients. A 2011 study reported a 73.8% reduction in red blood cell (RBC) glutathione in 12 T2DM patients (1.78 ± 0.28 vs. 6.75 ± 0.47 µmol/g Hb, *P* < 0.001) alongside lower plasma cysteine levels, attributed to impaired synthesis and elevated oxidative stress.^10^ A 2014 study of 79 T2DM patients observed reduced cysteine and glutathione levels, with a strong correlation (r = 0.81, *P* = 0.001) and an inverse relationship with insulin resistance (HOMA-IR, r = -0.65, *P*< 0.05).^11^

 Additionally, a 2018 study of 16 T2DM patients showed lower glutathione levels (0.35 ± 0.30 vs. 0.90 ± 0.42 mmol/L, *P* < 0.01) and synthesis rates (0.50 ± 0.69 vs. 1.03 ± 0.55 mmol/L/day, *P* < 0.05), particularly in those with microvascular complications, suggesting a role for cysteine deficiency in exacerbating oxidative stress.^12^ These findings indicate that sulfur deficiency may impair PDI activity, potentially leading to misfolded insulin with compromised disulfide bonds.^13^

 Cysteine scarcity could also elevate reactive oxygen species (ROS), activating nuclear factor kappa-light-chain-enhancer of activated B cells (NF-κB), which may upregulate pro-inflammatory cytokines (e.g., TNF-α, IL-6).^14^ These cytokines could induce c-Jun N-terminal kinase (JNK)-mediated serine phosphorylation of insulin receptor substrate-1 (IRS-1), potentially disrupting PI3K-Akt signaling.^15,16^ Furthermore, reduced cysteine availability may impair mucin synthesis, weakening gut barrier integrity and possibly enabling lipopolysaccharide (LPS)-induced endotoxemia via toll-like receptor 4 (TLR4), which could amplify systemic inflammation.^17^ This framework offers a potential explanation for the coexistence of hyperinsulinemia and hyperglycemia in T2DM; misfolded endogenous insulin may accumulate but lack bioactivity due to reduced IR affinity, whereas exogenous insulin, with intact disulfide bonds, may retain functionality.^8^

 Supporting evidence includes in vitro studies where cysteine supplementation in hyperglycemic U937 monocytes restored glutamate-cysteine ligase expression and glutathione levels, suggesting a potential role in mitigating sulfur-dependent insulin dysfunction.^11^ A 2016 study noted impaired glutathione peroxidase activity and elevated malondialdehyde in T2DM, indicating increased glutathione consumption under oxidative stress, which may contribute to insulin misfolding.^16^ Synthetic insulin analogs with disrupted A6-A11 bonds exhibited 50-70% reduced IR affinity, while A7-B7 mutations impaired PI3K-Akt signaling, aligning with the proposed mechanism.^8 Conversely, engineering an additional disulfide bond enhanced insulin stability, underscoring the importance of sulfur-dependent bonds.^8,17^

 This hypothesis challenges conventional T2DM models by suggesting that insulin resistance may partly stem from defective insulin structure rather than solely post-receptor signaling defects. It proposes the gut-mitochondria-sulfur-insulin axis as a potential driver of pathogenesis, with sulfur deficiency possibly disrupting disulfide bond integrity and insulin function. However, this hypothesis remains speculative, as direct structural evidence of endogenous insulin misfolding in T2DM patients is limited due to technical challenges in isolating native insulin. Future studies employing high-resolution liquid chromatography-tandem mass spectrometry (LC-MS/MS) and Raman spectroscopy are needed to validate disulfide bond disruptions and confirm the role of sulfur metabolism in T2DM pathogenesis. This hypothesis aims to stimulate research into sulfur metabolism’s role in T2DM, potentially offering new avenues for understanding its pathogenesis.

## Competing Interests

 The authors declare that there are no conflicts of interest.

## Ethical Approval

 Not applicable.
